# Combined Liquid Chromatography–Tandem Mass Spectrometry Analysis of Progesterone Metabolites

**DOI:** 10.1371/journal.pone.0117984

**Published:** 2015-02-13

**Authors:** Maša Sinreih, Sven Zukunft, Izidor Sosič, Jožko Cesar, Stanislav Gobec, Jerzy Adamski, Tea Lanišnik Rižner

**Affiliations:** 1 Institute of Biochemistry, Faculty of Medicine, University of Ljubljana, Ljubljana, Slovenia; 2 Institute of Experimental Genetics, Genome Analysis Centre, Helmholtz Zentrum München, München, Germany; 3 Faculty of Pharmacy, University of Ljubljana, Ljubljana, Slovenia; 4 Lehrstuhl für Experimentelle Genetik, Technische Universität München, Freising-Weihenstephan, Germany; 5 German Center for Diabetes Research, Neuherberg, Germany; Moffitt Cancer Center, UNITED STATES

## Abstract

Progesterone has a number of important functions throughout the human body. While the roles of progesterone are well known, the possible actions and implications of progesterone metabolites in different tissues remain to be determined. There is a growing body of evidence that these metabolites are not inactive, but can have significant biological effects, as anesthetics, anxiolytics and anticonvulsants. Furthermore, they can facilitate synthesis of myelin components in the peripheral nervous system, have effects on human pregnancy and onset of labour, and have a neuroprotective role. For a better understanding of the functions of progesterone metabolites, improved analytical methods are essential. We have developed a combined liquid chromatography—tandem mass spectrometry (LC-MS/MS) method for detection and quantification of progesterone and 16 progesterone metabolites that has femtomolar sensitivity and good reproducibility in a single chromatographic run. MS/MS analyses were performed in positive mode and under constant electrospray ionization conditions. To increase the sensitivity, all of the transitions were recorded using the Scheduled MRM algorithm. This LC-MS/MS method requires small sample volumes and minimal sample preparation, and there is no need for derivatization. Here, we show the application of this method for evaluation of progesterone metabolism in the HES endometrial cell line. In HES cells, the metabolism of progesterone proceeds mainly to (20S)-20-hydroxy-pregn-4-ene-3-one, (20S)-20-hydroxy-5α-pregnane-3-one and (20S)-5α-pregnane-3α,20-diol. The investigation of possible biological effects of these metabolites on the endometrium is currently undergoing.

## Introduction

Progesterone is a steroid hormone that is synthesized mainly in the ovaries, placenta and adrenal glands. In the human body, progesterone has a number of important functions. It is known to be involved in differentiation of endometrium [1], proliferative changes of breast glandular tissue that occur during the menstrual cycle [2], and maintenance of pregnancy and lactation [1], while also showing neuroprotective effects [3]. Elevated levels of progesterone during gestation provide protection against oxidative stress and immune reactions to the fetus, and have a role in the normal development of neurons [4]. Progesterone also appears to modulate bone remodeling, and thus to protect against bone loss [1]. While the role of progesterone in human is well known, the possible actions and implications of progesterone metabolites are still to be determined.

There is a growing body of evidence, that many metabolites are not inactive, but have significant biological effects [5–13]. A variety of progesterone metabolites has been identified in human cell lines, biological fluids, and tissues, including plasma [5,14,15], cerebrospinal fluid [15], and breast [9,15], endometrium [2], kidney [16], liver [17] and adipose [18] tissues. The 5α-pregnanes are known ligands for the GABAA receptor, and have anesthetic, anxiolytic and anticonvulsant activities [7]. Menstrual-cycle-related changes in mood, cognitive function, and drug sensitivity have been attributed to fluctuations of these 5α-pregnanes and their modulation of the GABA system. Changes in circulating levels of these metabolites are also associated with fatigue in patients with chronic liver disease [5] and chronic fatigue syndrome [7], and might also be involved in the pathogenesis of premenstrual dysphoric disorder [8]. Progesterone metabolites, and especially 3α-hydroxy-5α-pregnane-20-one, can promote neuroprotection, and progesterone as a prodrug can contribute to the repair of central nervous system injuries like stroke and traumatic brain injury [4]. Furthermore, progesterone metabolites have weak affinity for human mineralocorticoid receptors and can demonstrate either agonistic or antagonistic effects, although the main role of progesterone metabolism in the kidney, especially during pregnancy, is the protection of the mineralocorticoid receptor against high progesterone concentrations [19]. Progesterone metabolites also facilitate synthesis of myelin components in the peripheral nervous system, and thus they represent potential therapy options in demyelinating diseases, diabetic neuropathy, and peripheral nerve injury, among others [6]. However, the exact nature of these metabolites has yet to be determined. In breast cell lines it has been shown that 5α-pregnane-3,20-dione stimulates cell proliferation and detachment while (20S)-20-hydroxy-pregn-4-ene-3-one (20α-hydroxy-pregn-4-ene-3-one) suppresses cell proliferation and stimulates attachment. The proliferation of breast cancer cells appears to be related to the increased concentration of cancer-promoting 5α-pregnane metabolites [9,10]. The 5α/β-reduced metabolites might also have effects on human pregnancy [11,12] and onset of labour [13]. These metabolites might also provide protection against acute hypoxic stress and might affect pain perception in mother and fetus [11].

**Fig 1:**
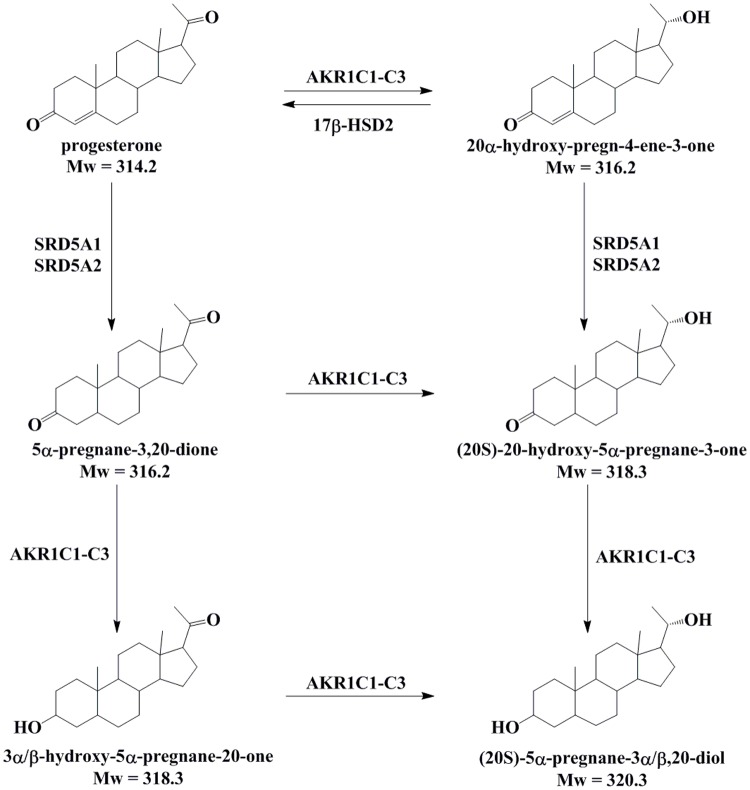
Proposed progesterone metabolism in human endometrium. Progesterone is metabolized by 20-ketosteroid reductases and 5α-reductases, to form (20S/R)-pregn-4-ene-3α,20-diol and 5α-pregnane-3,20-dione. Reductions of the 3-keto and 20-keto groups are catalysed by the aldo-keto reductases AKR1C1-AKR1C3. Here, AKR1C1-AKR1C3 can act on the 20-keto group of pregn-4-enes, and they can reduce both 3-keto and 20-keto groups of 5α-pregnanes. The progesterone (20S)-pregn-4-ene-3α,20-diol formed by the AKR1C enzymes can be oxidized back to progesterone by the 20α-hydroxysteroid dehydrogenase activity of 17β-hydroxysteroid dehydrogenase type 2 (HSD17B2). The formation of 5α-pregnanes is irreversible and is catalysed by 5α-reductases types 1 (SRD5A1) and 2 (SRD5A2) [35–37].

Progesterone is metabolized by reductions and conjugations, and its metabolites that are formed in different tissues vary. Progesterone is metabolized by 20-ketosteroid and 5α-reductases, to form 20α/β-hydroxy-pregn-4-ene-3-one and 5α-pregnane-3,20-dione. Reductions of 3-keto and 20-keto groups are catalyzed by the aldo-keto reductases, AKR1C1-AKR1C3 [20]. AKR1C1-AKR1C3 can act on the 20-keto group of pregn-4-enes and reduce both 3-keto and 20-keto groups of 5α-pregnanes [21]. The progesterone metabolite (20S)-20-hydroxy-pregn-4-ene-3-one thus formed can be oxidized back to progesterone by the 20α-hydroxysteroid dehydrogenase activity of 17β-hydroxysteroid dehydrogenase type 2 (HSD17B2) [22]. The formation of 5α-pregnanes is irreversible and is catalyzed by 5α-reductases types 1 and 2 (SRD5A1 and SRD5A2) (Fig. 1). Further metabolism is catalyzed by glucuronidases and sulfatases, to form the corresponding conjugates.

The strategies currently available for the analysis of progesterone metabolites include immunoassays [23], one or two dimension thin-layer chromatography [2,24,25], high pressure liquid chromatography (HPLC) [26,27], gas chromatography/mass spectrometry (GC/MS) [14,28,29], liquid chromatography/mass spectrometry (LC/MS) [30] and liquid chromatography—tandem mass spectrometry (LC-MS/MS). The reliability of immunoassays has been questioned due to the high probability of cross-reactivity between different stereoisomers, and it also requires large sample volumes, especially when a steroid is present at low concentrations [31,32]. The GC/MS and LC-MS/MS techniques are superior to immunoassays regarding specificity and sensitivity and have good linearity [33]. These methods require much smaller sample volumes and enable simultaneous analysis of multiple target analytes within one single run. While GC/MS methods require derivatization of the sample, only a minimal sample preparation is needed for LC-MS/MS techniques [33].

We have developed a LC-MS/MS method for detection and quantification of progesterone and 16 progesterone metabolites, with high sensitivity (detection in the femtomolar range), in a single chromatographic run. We demonstrate the utility of this approach for the analysis of progesterone metabolism in an epithelial endometrial cell line (HES cells). The endometrium is one of the most hormonally responsive tissues in the body, and its progesterone metabolism has not yet been studied in detail. Aberrant actions of progesterone can lead to neoplastic changes, including hyperplasia and endometrial cancer [34], and also to development of endometriosis [15] which calls for detailed understanding of progesterone metabolism in normal and diseased endometrial tissues and the corresponding model cell lines.

This established LC-MS/MS method has a potential to also be adapted for targeted metabolomics studies in model cell lines, as well as in plasma, urine and cerebrospinal fluid, and in tissue biopsies.

## Materials and Methods

### Reagents and chemicals

All chemicals and solvents were of the highest purity available. The commercial standards of testosterone, 3α-hydroxy-pregn-4-ene-20-one, 3β-hydroxy-pregn-4-ene-20-one, (20S)-20-hydroxy-pregn-4-ene-3-one, (20R)-20-hydroxy-pregn-4-ene-3-one (20β-hydroxy-pregn-4-ene-3-one), 5α-pregnane-3,20-dione, 3α-hydroxy-5α-pregnane-20-one, 3β-hydroxy-5α-pregnane-20-one, (20S)-20-hydroxy-5α-pregnane-3-one (20α-hydroxy-5α-pregnane-3-one), (20R)-20-hydroxy-5α-pregnane-3-one (20β-hydroxy-5α-pregnane-3-one), (20S)-5α-pregnane-3α,20-diol (5α-pregnane-3α,20α-diol), (20S)-5α-pregnane-3β,20-diol (5α-pregnane-3β,20α-diol), (20R)-5α-pregnane-3α,20-diol (5α-pregnane-3α,20β-diol) were from Steraloids (Newport, RI, USA). Progesterone, formic acid, Dulbecco’s modified Eagle’s medium (DMEM), fetal bovine serum, and trypsin-EDTA solution were from Sigma-Aldrich Chemie GmbH (Deisenhofen, Germany). Solvents were obtained from Carlo Erba Reagents (Rodano, Italy), Carl Roth GmbH (Karlsruhe, Germany), Sigma-Aldrich Chemie GmbH (Deisenhofen, Germany), Thermo Fisher Scientific (Geel, Belgium) and Alfa Aesar (Heysham, Lancashire, UK).

### Experimental procedure for preparation of pregn-4-ene-3,20-diols

These reactions were carried out under an argon atmosphere with magnetic stirring. Aluminium isopropoxide (2.60 g, 12.8 mmol) was added to a solution of progesterone (1.00 g, 3.2 mmol) in 40 mL freshly distilled 2-propanol. The reaction flask was equipped with a Vigreux column and refluxed until acetone was no longer detected in the distillate, by staining with 2,4-dinitrophenylhydrazine (for 8 h). After the reaction was complete, the solvent was evaporated; diethyl ether (50 mL) was added to the residue, followed by extraction with 2 M NaOH (2× 30 mL) and 10% aqueous citric acid. The organic layer was dried over Na_2_SO_4_, filtered, and evaporated under reduced pressure, to yield a crude mixture of four diastereoisomers (910 mg). HPLC analysis at 206 nm showed that the mixture contained four peaks that belonged to different products, while at 240 nm, no peak of the starting material (progesterone) was present. After separation of the mixture, and isolation and characterization of the products, this was revealed to contain 16.9% (20S)-pregn-4-ene-3β,20-diol (pregn-4-ene-3β,20α-diol), 7.4% (20S)-pregn-4-ene-3α,20-diol (pregn-4-ene-3α,20α-diol), 52.2% (20R)-pregn-4-ene-3β,20-diol (pregn-4-ene-3β,20β-diol) and 23.5% (20R)-pregn-4-ene-3α,20-diol (pregn-4-ene-3α,20β-diol).

### Purification and characterization of the pregn-4-ene-3,20-diols

The mixture of stereoisomers was first separated by reversed-phase flash chromatography on an Isolera One automated purification system (Biotage) that was equipped with variable dual-wavelength and an automatic fraction collector, employing the manufacturer’s proprietary C18 flash cartridges (SNAP KP-C18-HS 120 g). The mixture was dissolved in minimal amount of methanol, put on a samplet packed with C18 silica and dried. Fractions were eluted using 40 mL/min flow rate and water/ acetonitrile/ methanol gradient. Gradient conditions: solvent A: water/ acetonitrile = 50/ 50, solvent B: methanol; eluent: 100% A for initial 6 column volume, then linear gradient from 0% B to 100% B over next 12 column volumes. Fractions were collected based on the UV signal at 206 nm. The composition of individual fractions was first checked by analytical HPLC and fractions with similar composition were combined. Organic solvent was evaporated under reduced pressure and the aqueous residue was frozen in liquid nitrogen and lyophilized. In this manner, considerable amounts of pure (20S)-pregn-4-ene-3β,20-diol (first peak) and (20R)-pregn-4-ene-3β,20-diol (third peak) were isolated. However, some of the (20S)-pregn-4-ene-3α,20-diol (second peak) always eluted within the first and third peaks, or within both of these peaks, while some of the (20R)-pregn-4-ene-3α,20-diol (fourth peak) eluted with some of the third peak. The fraction containing the second peak and those containing the fourth peak were combined, the solvents removed, and the residues obtained were further purified by preparative HPLC. In this manner, all four stereoisomers were obtained in sufficiently pure forms for further studies. The amounts isolated varied from 18 mg for (20S)-pregn-4-ene-3α,20-diol, to 240 mg for (20R)-pregn-4-ene-3β,20-diol. However, these yields were not optimized, as we primarily focused on obtaining pure derivatives and, therefore, considerably more effort was put into isolation of the smallest peak (second peak) than for the other three peaks.

The HPLC analyses and separations were run on an Agilent 1100 system equipped with a quaternary pump, a multiple wavelength UV detector, and an automated fraction collector. Analytical HPLC was performed using an Agilent EclipsePlus C18 column (4.6 × 150 mm, 5 μm), with the mobile phase of water and acetonitrile (45:55; v/v), a 1.0 mL/min flow rate, 206 nm detection wavelength, and 25°C column temperature. Preparative HPLC was run on a BIA Eurospher C18 column (16.0 × 250 mm, 5 μm) with the eluent of water and acetonitrile (40:60; v/v), pre-heated to 40°C. The injection volume was 300 μL, the flow rate was 7.0 mL/min, the column temperature was 40°C, and fraction collection was triggered by detector signals at 206 nm. ^1^H NMR and ^13^C NMR spectra were recorded on a Bruker Avance 400 DPX at 302 K, and are reported in ppm using the solvent as internal standard. The coupling constants (*J*) are given in Hz, and the splitting patterns are designated as: s, singlet; bs, broad singlet; d, doublet; dd, double doublet; t, triplet; sym m, symmetrical multiplet; br m, broad multiplet; and m, multiplet. Mass spectra data and high-resolution mass measurements were performed on a VG-Analytical Autospec Q mass spectrometer.

### (20S)-pregn-4-ene-3β,20-diol

^1^H NMR (400 MHz, CDCl_3_) *δ* 0.66 (s, 3H, 18-CH_3_), 0.69–0.77 (m, 1H), 0.81–0.90 (m, 1H), 0.91–0.98 (m, 1H), 1.03 (s, 3H, 19-CH_3_), 1.06–1.18 (m, 2H), 1.20 (d, *J* = 6.15 Hz, 3H, 21-CH_3_), 1.21–1.25 (m, 1H), 1.27–1.33 (m, 2H), 1.34–1.56 (m, 6H), 1.61–1.75 (m, 3H), 1.82–1.97 (m, 3H), 1.98–2.03 (m, 1H), 2.13–2.22 (m, 1H), 3.69 (sym m, 1H, 20-H), 4.09–4.17 (br m, 1H, 3-H), 5.26 (dt, *J* = 1.8, 1.6 Hz, 1H, 4-H); ^13^C NMR (100 MHz, CDCl_3_) *δ* 12.47, 18.81, 20.62, 23.37, 24.02, 25.55, 29.40, 32.07, 32.95, 35.26, 35.45, 37.23, 38.74, 41.63, 54.28, 55.86, 58.30, 67.86, 70.24, 123.30, 147.45; HRMS (ESI) m/z calculated for C_21_H_33_O_2_ [M–H]^–^ 317.2481, found 317.2484; HPLC purity: 96.4%; retention time: 4.4 min (S1 Fig., S2 Fig., S3 Fig.)

### (20S)-pregn-4-ene-3α,20-diol

^1^H NMR (400 MHz, CDCl_3_) *δ* 0.69 (s, 3H, 18-CH_3_), 0.80–0.88 (m, 1H), 0.90–0.97 (m, 1H), 0.98 (s, 3H, 19-CH_3_), 0.99–1.05 (m, 1H), 1.09–1.20 (m, 2H), 1.22 (d, *J* = 6.15 Hz, 3H, 21-CH_3_), 1.29–1.39 (m, 3H), 1.40–1.47 (m, 1H), 1.48–1.52 (m, 4H), 1.58–1.61 (m, 1H), 1.63–1.77 (m, 4H), 1.85–1.96 (m, 2H), 2.00–2.07 (m, 1H), 2.17–2.27 (m, 1H), 3.71 (sym m, 1H, 20-H), 4.04–4.10 (br m, 1H, 3-H), 5.46 (dd, *J* = 5.0, 1.8 Hz, 1H, 4-H); ^13^C NMR (100 MHz, CDCl_3_) *δ* 12.18, 17.70, 20.82, 23.09, 23.75, 25.24, 27.45, 31.28, 31.97, 32.36, 35.04, 37.15, 38.50, 41.39, 53.65, 55.53, 58.01, 63.85, 69.94, 120.32, 149.83; HRMS (ESI) m/z calculated for C_21_H_33_O_2_ [M–H]^–^ 317.2481, found 317.2488; HPLC purity: 99.3%; retention time: 6.1 min (S4 Fig., S5 Fig., S6 Fig.).

### (20R)-pregn-4-ene-3β,20-diol

^1^H NMR (400 MHz, CDCl_3_) *δ* 0.68–0.73 (m, 1H), 0.74 (s, 3H, 18-CH_3_), 0.80–0.91 (m, 1H), 0.93–1.01 (m, 1H), 1.03 (s, 3H, 19-CH_3_), 1.10 (d, *J* = 6.15 Hz, 3H, 21-CH_3_), 1.12–1.19 (m, 2H), 1.20–1.26 (m, 2H), 1.26–1.33 (m, 2H), 1.34–1.43 (m, 4H), 1.49–1.55 (m, 1H), 1.58–1.70 (m, 4H), 1.88–1.95 (m, 1H), 1.95–2.06 (m, 2H), 2.12–2.22 (m, 1H), 3.70 (sym m, 1H, 20-H), 4.08–4.16 (br m, 1H, 3-H), 5.25 (dt, *J* = 1.8, 1.6 Hz, 1H, 4-H); ^13^C NMR (100 MHz, CDCl_3_) *δ* 12.53, 18.96, 20.94, 23.69, 24.51, 25.65, 29.54, 32.23, 33.17, 35.43, 35.82, 37.40, 39.98, 42.45, 54.49, 55.67, 58.50, 68.00, 70.60, 123.42, 147.63; HRMS (ESI) m/z calculated for C_21_H_33_O_2_ [M–H]^–^ 317.2481, found 317.2485; HPLC purity: 96.4%; retention time: 6.5 min (S7 Fig., S8 Fig., S9 Fig.).

### (20R)-pregn-4-ene-3α,20-diol

^1^H NMR (400 MHz, CDCl_3_) *δ* 0.73 (s, 3H, 18-CH_3_), 0.76–0.81 (m, 1H), 0.82–0.93 (m, 2H), 0.94 (s, 3H, 19-CH_3_), 0.96–1.01 (m, 1H), 1.09 (d, *J* = 6.15 Hz, 3H, 21-CH_3_), 1.11–1.31 (m, 4H), 1.33–1.40 (m, 4H), 1.42–1.54 (m, 4H), 1.57–1.61 (m, 1H), 1.65–1.71 (m, 2H), 1.95–2.05 (m, 2H), 2.12–2.22 (m, 1H), 3.67 (sym m, 1H, 20-H), 4.00–4.06 (br m, 1H, 3-H), 5.41 (dd, *J* = 5.0, 1.8 Hz, 1H, 4-H); ^13^C NMR (100 MHz, CDCl_3_) *δ* 12.46, 18.08, 21.37, 23.63, 24.46, 25.59, 27.83, 31.65, 32.35, 32.79, 35.63, 37.54, 39.96, 42.44, 54.06, 55.56, 58.46, 64.22, 70.54, 120.66, 150.22; HRMS (ESI) m/z calculated for C_21_H_33_O_2_ [M–H]^–^ 317.2481, found 317.2489; HPLC purity: 97.4%; retention time: 8.8 min (S10 Fig., S11 Fig., S12 Fig.).

### Cell culture

The HES human endometrial cell line was used, which was established from benign proliferative endometrium [27]. The HES cell line was a gift from the Laboratory of Perinatal Research at the Ohio State University. Cells were cultured in DMEM containing 10% fetal bovine serum, without any antibiotics, in a humidified atmosphere of 5% CO_2_ and 95% air at 37°C. The HES cells were passaged at a 1:4 dilution.

### Experimental design

The HES cells were plated into 6-well plates at a density of 10^6^ cells per well. 35^th^ passage of cells was used. To avoid potential steroid-mimicking effects, the media was replaced after 24 h with serum-free and phenol-red-free medium, and progesterone at a final concentration of 500 nM in dimethyl sulfoxide was added, giving the final dimethyl sulfoxide concentration of 0.25%. The cells were incubated with progesterone for 4, 8 or 24 h, or without progesterone for 24 h (for observation of matrix effects). Medium from two wells of 6-well plates was combined and ethyl acetate was added, the mixture was vortexed for 3 min. After centrifugation, the upper ethyl acetate phase was collected, the solvent was evaporated off, and the steroids were dissolved in 60 μL of a mixture of acetonitrile/ water/ formic acid (50/50/0.5; v/v) and testosterone (100 ng/mL).

### LC-MS/MS

Chromatographic separations were performed on an Agilent Infinity 1260 HPLC system using a Kinetex 2.6μ XB-C18 column (150 × 4.6 mm; Phenomenex, Aschaffenburg, Germany), equipped with a Securityguard guard column and Securityguard cartridges (C18; 4 × 3.0 mm; Phenomenex, Aschaffenburg, Germany). Samples of 20 μL were injected via a PAL HTC-*xt* autosampler (CTC Analytics, Zwingen, Switzerland). The mobile phase consisted of (A) 5% acetonitrile, 0.5% formic acid in water, and (B) 0.5% formic acid in acetonitrile, and the flow rate was 690 μL/min. The column temperature was 25°C. The gradient elution was: initial condition, 0.0–10.0 min, 60% A; 10.0–25.0 min, 60%–35% A; 25.0–30.0 min, 35% A; 30.0–30.1 min, 35%–60% A; 30.1–40.0 min equilibration at 60% A. The HPLC was coupled to a QTRAP 5500 System (AB Sciex Deutschland GmbH, Darmstadt, Germany), with all controlled by the Analyst 1.6 software (AB Sciex Deutschland GmbH, Darmstadt, Germany). MS/MS analysis was performed in positive ion mode and with constant electrospray ionization (ESI) conditions. Source-dependent parameters were determined by flow injection analysis, and were as follows: curtain gas, 20; collision gas, medium; ion spray voltage, 5500 V; source temperature, 400°C; ion source gas 1, 60; ion source gas 2, 80. The compound-dependent parameters (declustering potential, entrance potential, collision energy, collision cell exit potential) were optimized by direct infusion of individual standard solutions at concentration ranging from 100 ng/mL to 1000 ng/mL. All transitions were recorded using the Scheduled MRM algorithm with the purpose of increasing sensitivity. The target scan time was set to 10 s, with an MRM detection window of 70 s. The resolution for first and third quadrupole (Q1 and Q3) was set as a unit; the pause between mass ranges was set at 5 ms.

The concentration of each steroid was calculated on the basis of standard curves. Calibration curves were constructed from seven concentrations, covering the range from 2 ng/mL to 500 ng/mL. Testosterone was added to each sample at a final concentration of 100 ng/mL, and used as internal standard.

### Recovery calculations and inter-day variability

Replicas of samples (n = 5) without and with the addition of the authentic compounds were analysed by LC-MS/MS. Mean, standard deviation, and coefficient of variation were calculated (Table 1). The recovery (R) was calculated as the mean of the measured progesterone metabolite concentration with standard addition (M_+_) divided by the mean of the expected metabolite concentration (E), while E was calculated as the mean of the measured metabolite concentration without standard addition (M_-_) plus the standard amount added (AA), as shown in Equation (1):

$$R\;=\;\bar{M_{+}}\;/\;\bar{E};\;\bar{E}\;=\;\bar{M_{-}}\;+\;AA$$ (1)

All measurements were repeated after three days and inter-day variability was calculated.

**Table 1:** Recovery calculations (n = 5) before addition of individual steroids.

Steroid	Measured concentration before addition (ng/mL)			Inter-day variability
	mean	S. D.	CV (%)	CV (%)
(20S)-Pregn-4-ene-3β,20-diol	9.0	0.2	2.3	9.7
(20S)-20-Hydroxy-pregn-4-ene-3-one	ND			
(20S)-5α-Pregnane-3β,20-diol	25.0	1.2	4.8	3.9
(20S)-Pregn-4-ene-3α,20-diol	ND			
(20R)-Pregn-4-ene-3β,20-diol	ND			
(20R)-20-Hydroxy-pregn-4-ene-3-one	ND			
(20S)-5α-Pregnane-3α,20-diol	327.0	12.2	3.7	4.4
Progesterone	2.9	0.2	5.2	6.1
(20S)-20-Hydroxy-5α-pregnane-3-one	2.0	0.2	12.2	15.2
(20R)-Pregn-4-ene-3α,20-diol	ND			
3α-Hydroxy-pregn-4-ene-20-one	ND			
3β-Hydroxy-pregn-4-ene-20-one	ND			
3β-Hydroxy-5α-pregnane-20-one	ND			
(20R)-5α-Pregnane-3α,20-diol	ND			
3α-Hydroxy-5α-pregnane-20-one	18.4	0.8	4.5	4.9
(20R)-20-Hydroxy-5α-pregnane-3-one	ND			
5α-Pregnane-3,20-dione	ND			

ND, not detected

S.D., standard deviation

CV, coefficient of variance

### Matrix effects

The post column infusion method was used to visualize adverse matrix effects. A solution containing tested metabolites with or without internal standard (100 ng/mL of each progesterone metabolite) was infused at 10 μL/min using a syringe pump assembled onto the mass spectrometer. A sample of blank matrix (HES cells incubated for 24 h in medium without progesterone; extraction as described above) was injected onto the LC column. The effluent from the LC column thus combined with the permanent infused standards entered the mass spectrometer for assessment of matrix effects. The analyses were repeated two times with internal standards and four times without.

## Results and Discussion

### Synthesis of pregn-4-ene-3,20-diols

**Fig 2:**
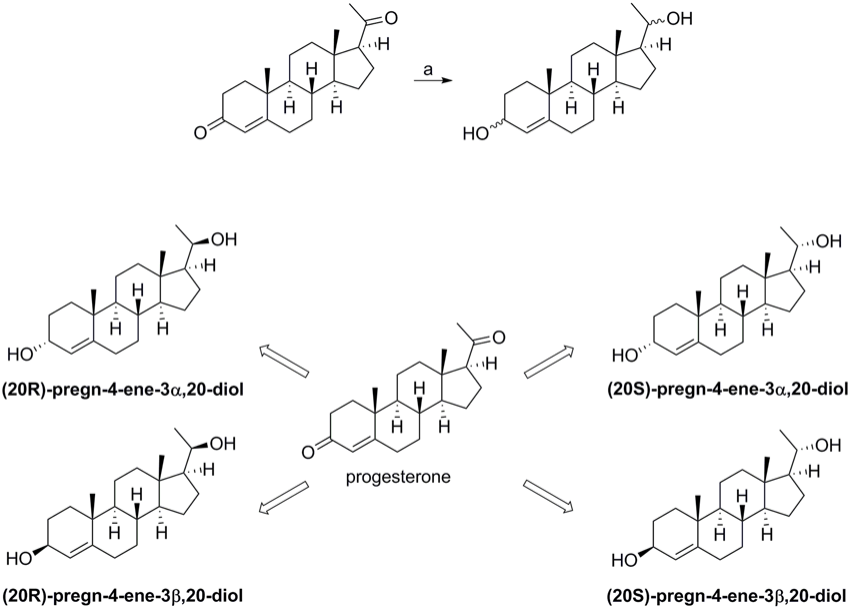
Reaction scheme and structural formulae of the pregn-4-ene-metabolites. Reagents and conditions: (a) Al(O-*i*-Pr)_3_, 2-propanol, reflux.

The reduction of progesterone was performed as described earlier by Wiebe et al., with slight modifications [29]. Carbonyls at the allylic C-3 and at C-20 were reduced using aluminum isopropoxide in 2-propanol (Fig. 2), to obtain a mixture of four steroisomers (pregn-4-ene-3,20-diols). Individual diols were subsequently isolated by reverse-phase separation of the mixture, using flash chromatography followed by preparative HPLC.

The exact structures of the individual isolated derivatives were assigned by comparison of their recorded spectroscopic data with those previously published [29]. As described, the chemical shifts of the characteristic signals (specially the methyl groups at positions 18, 19 and 21) and the specific splitting patterns allowed unambiguous assignment of the stereochemistry of both hydroxyl groups in each of the four pregn-4-ene-3,20-diols (Fig. 2).

### Optimization of MS/MS conditions

The retention times and monitoring transitions for progesterone and 16 of its metabolites used in the assay are given in Table 2. The mass of the formed precursor ions was determined by direct infusion into the ion source. [M+H]^+^, [M-H_2_O+H]^+^ or [M-2H_2_O+H]^+^ were selected. During the method development, two SRM transitions between the precursor ion and the two most abundant fragment ions were monitored for each steroid, to confirm the identity of the target compound, although in the final method, only the transition with the best response for each compound was monitored. As mentioned above, all of the transitions were monitored using the Scheduled MRM algorithm. With this option, transitions are measured around the expected retention time. Scheduled MRM decreases the number of concurrent transitions, which allows both the cycle time and the dwell time to be automatically optimized for the highest sensitivity, accuracy and reproducibility.

**Table 2:** Selected reaction monitoring transitions and the optimum LC-MS/MS conditions for the internal standard testosterone, progesterone and its metabolites.

Number	Steroid	Q1 (Da)	Q3 (Da)	Rt (min)	DP (Volts)	EP (Volts)	CE (Volts)	CXP (Volts)
1	Testosterone	289.2 [M+H]^+^	109.2	9.2	120	10	47	23
2	(20S)-Pregn-4-ene-3β,20-diol	301.1 [M-H_2_O+H]^+^	79.1	15.4	70	12	60	28
3	(20S)-20-Hydroxy-pregn-4-ene-3-one	317.1 [M+H]^+^	96.9	17.6	70	10	20	16
4	(20S)-5α-Pregnane-3β,20-diol	285.1 [M-2 H_2_O+H]^+^	66.9	18.2	110	8	30	10
5	(20S)-Pregn-4-ene-3α,20-diol	301.1 [M-H_2_O+H]^+^	79.1	19.4	70	12	60	28
6	(20R)-Pregn-4-ene-3β,20-diol	301.1 [M-H_2_O+H]^+^	77.0	19.8	70	12	80	28
7	(20R)-20-Hydroxy-pregn-4-ene-3-one	299.2 [M-H_2_O+H]^+^	81.0	21.7	90	10	20	10
8	(20S)-5α-Pregnane-3α,20-diol	285.1 [M-2 H_2_O+H]^+^	78.9	22.7	110	8	30	10
9	Progesterone	315.1 [M+H]^+^	109.1	22.8	110	10	30	10
10	(20S)-20-Hydroxy-5α-pregnane-3-one	319.2 [M+H]^+^	91.0	22.9	150	8	60	10
11	(20R)-Pregn-4-ene-3α,20-diol	301.1 [M-H_2_O+H]^+^	77.0	23.2	70	12	80	28
12	3α-Hydroxy-pregn-4-ene-20-one	299.1 [M-H_2_O+H]^+^	81.0	23.3	90	10	20	10
13	3β-Hydroxy-pregn-4-ene-20-one	317.1 [M+H]^+^	96.9	23.8	70	10	20	16
14	3β-Hydroxy-5α-pregnane-20-one	319.2 [M+H]^+^	91.0	23.8	150	8	60	10
15	(20R)-5α-Pregnane-3α,20-diol	285.1 [M-2 H_2_O+H]^+^	78.9	25.9	110	8	30	10
16	3α-Hydroxy-5α-pregnane-20-one	319.2 [M+H]^+^	77.0	26.1	150	8	80	10
17	(20R)-20-Hydroxy-5α-pregnane-3-one	319.2 [M+H]^+^	77.0	27.0	150	8	80	10
18	5α-Pregnane-3,20-dione	317.1 [M+H]^+^	91.1	27.4	90	10	60	10

Q1, first quadrupole; Q3, third quadrupole; Rt, retention time

DP, declustering potential; EP, entrance potential; CE, collision energy; CXP, collision cell exit potential

### Optimization of chromatographic conditions

**Fig 3:**
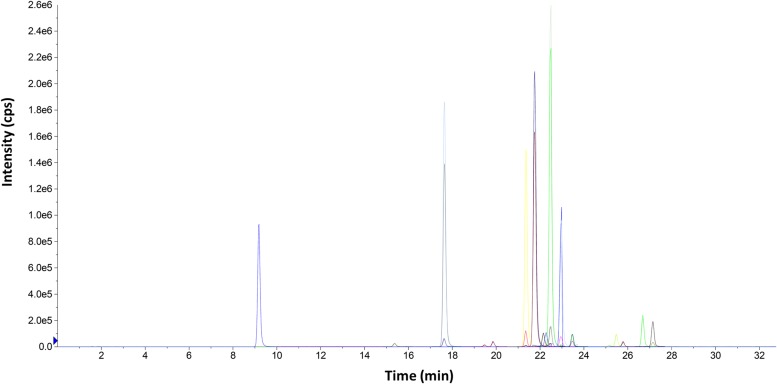
Representative chromatogram showing separation of progesterone and its metabolites. Representative extracted- ion chromatogram of a 100 ng/mL standard mixture of progesterone and its metabolites.

A C18 stationary phase (Kinetex 2.6μ XB-C18 column) was chosen for the separation of progesterone and its metabolites considering the wide use of this type of column for steroid separation and our previous experience in separation of progesterone metabolites. To optimize the chromatographic separation, different mobile phases were tested. The best separation was achieved with the combination of 5% acetonitrile in water and acetonitrile. The elution gradient and flow rate were adjusted to improve the chromatographic resolution, to obtain narrower chromatographic peaks, and to reduce the analysis time. It should be noted that 65% of the organic solvent was used for 5 min during the run, in order to clean the column and to avoid carry-over contamination. Different column oven temperatures were also tested (20°C, 25°C, 30°C, 35°C), where the peak shapes and chromatographic responses improved when 25°C was used. Later, to improve the sensitivity of the ESI, different buffers and acids were tested (ammonium acetate, formic acid, acetic acid). Formic acid was better than others as it improved the response of the progesterone metabolites. The effects of the formic acid concentration (0.1%, 0.2%, 0.3%, 0.5%, 0.8%) on the response of the progesterone metabolites were also investigated, with 0.5% formic acid providing the best response. Fig. 3 shows a representative chromatogram using this method.

### Quantification

**Fig 4:**
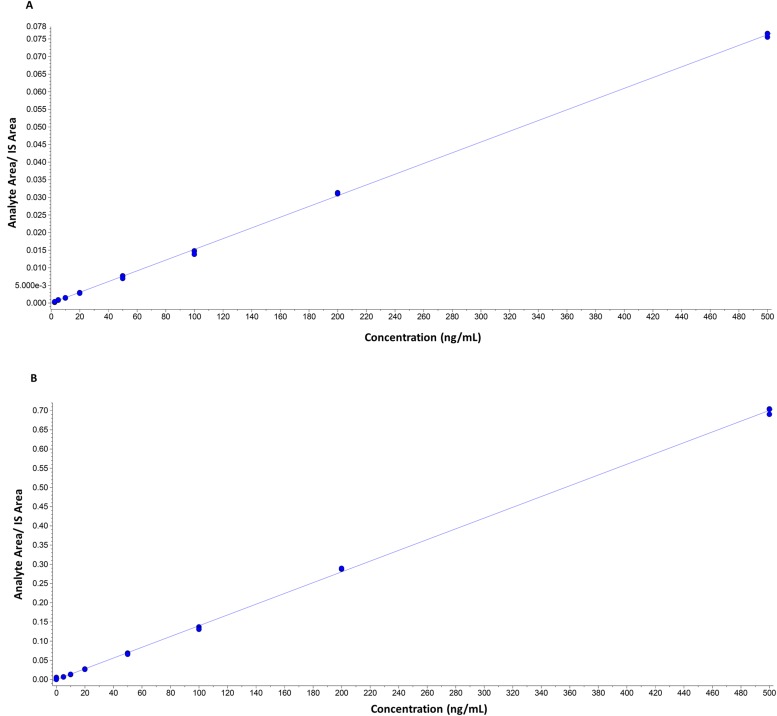
Representative calibration curves in solvent. A) (20S)-pregn-4-ene-3α,20-diol (r = 0.9999, y = 0.00015x). B) (20S)-5α-pregnane-3α,20-diol (r = 0.9998, y = 0.00014). Curves are constructed out of three measurements for each concentration.

**Fig 5:**
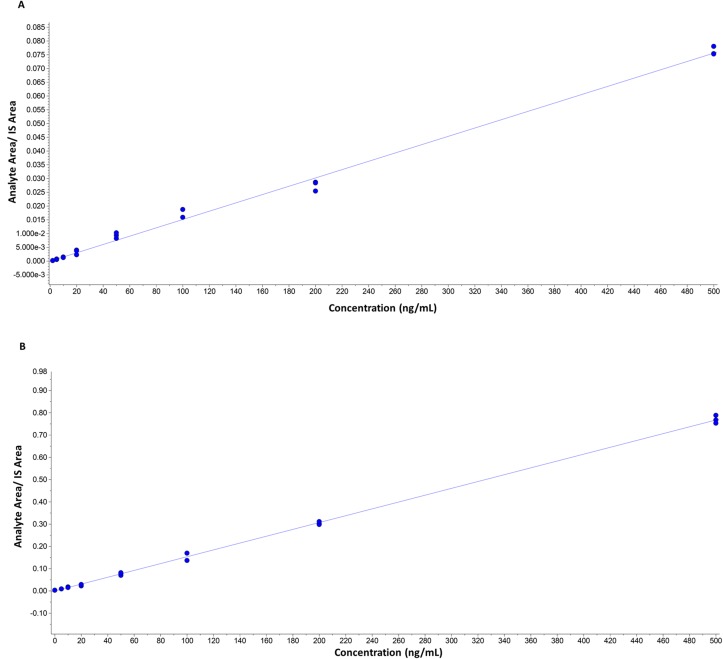
Representative calibration curves in matrix. A) (20S)-pregn-4-ene-3α,20-diol (r = 0.9985, y = 0.00015x). B) (20S)-5α-pregnane-3α,20-diol ((r = 0.9997, y = 0.00015). Curves are constructed out of three measurements for each concentration.

Quantification was based on linear regression calibration curves, using the internal standard approach. The linear responses covered the range from 2 ng/mL to 500 ng/mL. Calibration curves gave good fits (r^2^ >0.99). Representative calibration curves in solvent and in matrix are presented in Figs. 4 and 5.

### Precision, recovery an inter-day variability

To evaluate the precision and recovery, five replicate analyses of the same sample (HES, 24 h) were performed before and after addition of a known amount (10 ng/mL, 50 ng/mL and 250 ng/mL) of the metabolites. As shown in Tables 1, 3, 4 and 5, there was satisfactory precision in the analysis of all of the metabolites at the lower, middle and high limits of detection (coefficient of variance, <15%). The results of the recovery experiments were satisfactory (difference in the expected and measured concentrations <10%) for most of the metabolites, except for (20S)-pregn-4-ene-3α,20-diol, (20R)-pregn-4-ene-3α,20-diol and (20R)-pregn-4-ene-3α,20-diol after addition of 10 ng/mL of metabolites and 3α-hydroxy-pregn-4-ene-20-one after addition of 50 ng/mL of metabolites. The results of inter-day variability experiments were satisfactory (coefficient of variance, <10%) for most of the metabolites except for (20S)-20-hydroxy-5α-pregnane-3-one, (20S)-pregn-4-ene-3α,20-diol and (20R)-pregn-4-ene-3α,20-diol at low concentrations. The possibility of steroid contamination from the solvent and glassware was investigated by analysis of blank samples; there was no trace of metabolites in the blank samples (S13 Fig.).

**Table 3:** Recovery calculations (n = 5) after addition of 10 ng/mL of individual steroid.

Steroid	Measured concentration after addition of 10 ng/mL of individual steroid (ng/mL)			Recovery (%)		Inter-day variability
	mean	S. D.	CV (%)	mean	S.D.	CV (%)
(20S)-Pregn-4-ene-3β,20-diol	20.1	0.7	3.5	105.8	3.7	3.9
(20S)-20-Hydroxy-pregn-4-ene-3-one	10.3	0.5	4.9	103.3	5.1	4.5
(20S)-5α-Pregnane-3β,20-diol	35.9	1.1	3.0	102.8	3.0	3.6
(20S)-Pregn-4-ene-3α,20-diol	11.4	1.6	13.7	114.4	15.7	13.3
(20R)-Pregn-4-ene-3β,20-diol	11.1	0.9	8.2	111.0	9.1	6.0
(20R)-20-Hydroxy-pregn-4-ene-3-one	14.6	0.8	5.6	146.4	8.2	7.2
(20S)-5α-Pregnane-3α,20-diol	343.8	13.1	3.8	102.0	3.9	3.4
Progesterone	14.6	0.6	4.2	113.2	4.7	6.9
(20S)-20-Hydroxy-5α-pregnane-3-one	13.1	0.9	6.5	109.1	7.1	6.9
(20R)-Pregn-4-ene-3α,20-diol	11.7	1.1	9.2	116.9	10.8	7.8
3α-Hydroxy-pregn-4-ene-20-one	10.4	1.1	10.4	104.2	10.8	10.2
3β-Hydroxy-pregn-4-ene-20-one	10.4	0.6	5.7	103.5	5.9	5.3
3β-Hydroxy-5α-pregnane-20-one	9.8	0.4	4.3	97.7	4.2	3.6
(20R)-5α-Pregnane-3α,20-diol	10.1	0.6	5.5	100.6	5.5	8.2
3α-Hydroxy-5α-pregnane-20-one	30.1	1.4	4.8	106.0	5.0	4.3
(20R)-20-Hydroxy-5α-pregnane-3-one	10.0	0.5	4.8	100.0	4.8	4.8
5α-Pregnane-3,20-dione	10.7	0.7	6.3	106.7	6.7	6.9

S.D., standard deviation

CV, coefficient of variance

**Table 4:** Recovery calculations (n = 5) after addition of 50 ng/mL of individual steroid.

Steroid	Measured concentration after addition of 50 ng/mL of individual steroid (ng/mL)			Recovery (%)		Inter-day variability
	mean	S. D.	CV (%)	mean	S.D.	%
(20S)-Pregn-4-ene-3β,20-diol	63.5	2.4	3.8	107.7	4.1	3.2
(20S)-20-Hydroxy-pregn-4-ene-3-one	47.9	1.5	3.1	95.8	2.9	2.9
(20S)-5α-Pregnane-3β,20-diol	75.0	2.0	2.7	100.1	2.7	2.4
(20S)-Pregn-4-ene-3α,20-diol	43.0	1.2	2.8	86.0	2.4	4.4
(20R)-Pregn-4-ene-3β,20-diol	51.5	1.4	2.6	102.9	2.7	3.1
(20R)-20-Hydroxy-pregn-4-ene-3-one	69.1	2.1	3.0	138.2	4.1	2.6
(20S)-5α-Pregnane-3α,20-diol	372.2	10.5	2.8	98.7	2.8	2.6
Progesterone	57.8	2.4	4.1	109.3	4.5	5.7
(20S)-20-Hydroxy-5α-pregnane-3-one	53.8	2.1	3.8	103.5	4.0	4.3
(20R)-Pregn-4-ene-3α,20-diol	51.4	1.9	3.7	102.8	3.8	3.1
3α-Hydroxy-pregn-4-ene-20-one	52.0	1.5	2.9	104.2	10.8	6.6
3β-Hydroxy-pregn-4-ene-20-one	48.2	1.5	3.1	96.4	3.0	3.2
3β-Hydroxy-5α-pregnane-20-one	48.7	1.6	3.2	97.5	3.2	2.8
(20R)-5α-Pregnane-3α,20-diol	48.8	3.1	6.4	97.6	6.3	4.8
3α-Hydroxy-5α-pregnane-20-one	68.9	5.0	7.2	100.7	7.3	5.1
(20R)-20-Hydroxy-5α-pregnane-3-one	49.3	1.5	3.0	98.7	3.0	2.7
5α-Pregnane-3,20-dione	50.7	1.6	3.1	101.4	3.2	2.9

S.D., standard deviation

CV, coefficient of variance

**Table 5:** Recovery calculations (n = 5) after addition of 250 ng/mL of individual steroid.

Steroid	Measured concentration after addition of 250 ng/mL of individual steroid (ng/mL)			Recovery (%)		Inter-day variability
	mean	S. D.	CV (%)	mean	S.D.	(CV) %
(20S)-Pregn-4-ene-3β,20-diol	272.4	5.9	2.2	105.2	2.3	3.4
(20S)-20-Hydroxy-pregn-4-ene-3-one	226.8	8.3	3.7	90.7	3.3	3.5
(20S)-5α-Pregnane-3β,20-diol	265.6	9.0	3.4	96.6	3.3	3.4
(20S)-Pregn-4-ene-3α,20-diol	189.4	11.0	5.8	75.8	4.4	5.6
(20R)-Pregn-4-ene-3β,20-diol	247.6	11.7	4.7	99.0	4.7	4.3
(20R)-20-Hydroxy-pregn-4-ene-3-one	324.4	13.1	4.0	129.8	5.2	4.5
(20S)-5α-Pregnane-3α,20-diol	553.8	16.4	3.0	96.0	2.8	3.0
Progesterone	250.2	9.5	3.8	98.9	3.8	6.2
(20S)-20-Hydroxy-5α-pregnane-3-one	254.6	11.7	4.6	101.0	4.6	5.7
(20R)-Pregn-4-ene-3α,20-diol	249.2	9.4	3.8	99.7	3.7	4.3
3α-Hydroxy-pregn-4-ene-20-one	228.0	9.5	4.2	91.2	3.8	5.2
3β-Hydroxy-pregn-4-ene-20-one	222.8	9.4	4.2	89.1	3.8	3.8
3β-Hydroxy-5α-pregnane-20-one	242.6	13.9	5.7	97.0	5.6	5.0
(20R)-5α-Pregnane-3α,20-diol	225.4	10.4	4.6	90.2	4.1	6.1
3α-Hydroxy-5α-pregnane-20-one	258.0	11.7	4.5	96.1	4.3	4.6
(20R)-20-Hydroxy-5α-pregnane-3-one	246.8	12.9	5.2	98.7	5.1	5.1
5α-Pregnane-3,20-dione	250.8	12.0	4.8	100.3	4.8	5.0

S.D., standard deviation

CV, coefficient of variance

### Matrix effects

Matrix effects were evaluated by post-column infusion of the mixture of progesterone metabolites at 100 ng/mL with or without internal standard. No matrix effects (indicated by ionization suppression or enhancement) were observed throughout the entire sample run window in the presence of matrix component, confirming that the matrix effect did not impact the established assay method.

### Results of metabolism of progesterone in HES cells, and proposed model of metabolism

**Fig 6:**
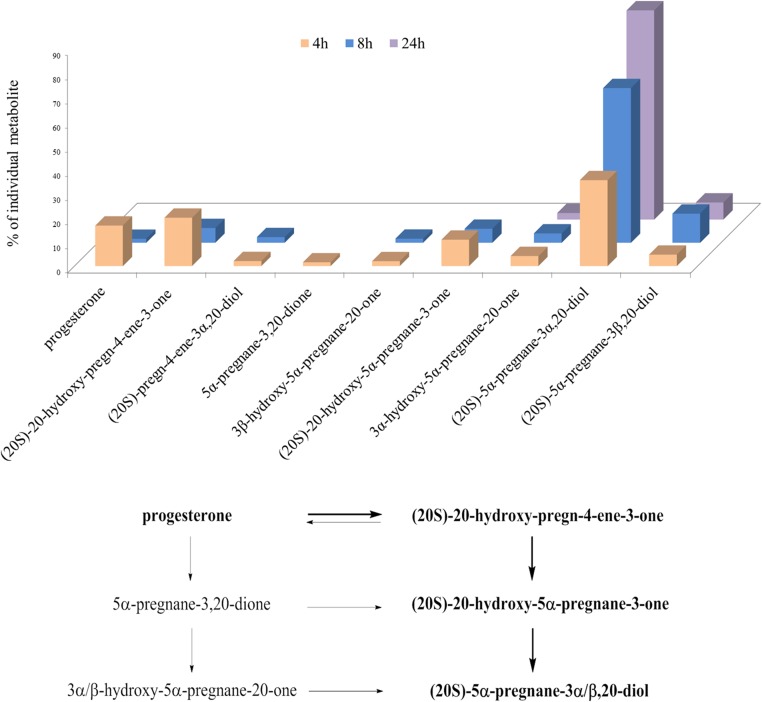
Progesterone metabolites formed in the HES cell line. Histogram representing the percentages of progesterone and its most abundant metabolites detected at different times. The scheme of the proposed metabolism is shown below. The main pathway is marked in **bold**. First, the 20-keto group of progesterone is reduced to form (20S)-20-hydroxy-pregn-4-ene-3-one, which is reduced at position C5 to form (20S)-20-hydroxy-5α-pregnane-3-one. Then (20S)-20-hydroxy-5α-pregnane-3-one is further metabolized at the 3-keto group, to form mainly (20S)-5α-pregnane-3α,20-diol, and also (20S)-5α-pregnane-3β,20-diol to a lesser extent.

The HES cell line was established from benign proliferative endometrium [27], and thus it represents a good model for establishing and testing a method for the detection and quantification of progesterone metabolites in normal and diseased human endometrium. The metabolism of progesterone in HES cells proceeds very quickly. After 4 h of incubation, we detected only a small fraction of the added progesterone. Progesterone was metabolized mainly to (20S)-20-hydroxy-pregn-4-ene-3-one, (20S)-20-hydroxy-5α-pregnane-3-one, and (20S)-5α-pregnane-3α,20-diol. After an 8-h incubation, the most abundant metabolite was (20S)-5α-pregnane-3α,20-diol (64%), followed by (20S)-5α-pregnane-3β,20-diol (12%), (20S)-20-hydroxy-pregn-4-ene-3-one (6%) and (20S)-20-hydroxy-5α-pregnane-3-one (6%). After 24 h, three main metabolites were detected, (20S)-5α-pregnane-3α,20-diol (87%), (20S)-5α-pregnane-3β,20-diol (7%) and (20S)-20-hydroxy-5α-pregnane-3-one (3%) (Fig. 5). The proposed main pathway of progesterone metabolism in this cell line is as follows: first, the 20-keto group of progesterone is reduced, to form (20S)-20-hydroxy-pregn-4-ene-3-one, which is reduced at position C5, to form (20S)-20-hydroxy-5α-pregnane-3-one; this is further metabolized at the 3-keto group to form mainly (20S)-5α-pregnane-3α,20-diol, and (20S)-5α-pregnane-3β,20-diol to a lesser extent (Fig. 6).

In contrast to our results in these epithelial HES cells, Arici *et al* showed that in endometrial stromal and glandular cells, after 24 h, 100 nM progesterone is metabolized mainly to 3β-hydroxy-5α-pregnane-20-one, followed by 5α-pregnane-3,20-dione, and 3α-hydroxy-5α-pregnane-20-one, while no (20S)-20-hydroxy-pregn-4-ene-3-one was detected [25]. This discrepancy can be explained by different methodological approach (thin-layer chromatography *versus* LC-MS/MS) and different concentration of progesterone (100 nM *versus* 500 nM).

### Biological and clinical significance

Our method was developed for the measurement of progesterone metabolites in various model cell lines of normal and diseased tissues. The LC-MS/MS approach allows the identification and quantification of progesterone metabolites for which their biological roles are yet to be determined in individual tissues. Our method also opens up the possibility to measure progesterone metabolites in tissue samples or blood. This might be relevant in the future, as progesterone metabolites might represent novel biomarkers for a variety of neurological diseases, and also for traumatic brain injuries.

## Conclusions

We have described here a method for the separation, identification and quantification of progesterone metabolites in human cell lines. The major advantages of our LC-MS/MS method over other published methods are as follows: 1) the method covers a wide range of possible progesterone metabolites; 2) there is no need for derivatization, with the consequent significantly decreased sample preparation time; 3) all of the metabolites are separated in a single run and are unambiguously identified by MS/MS; and 4) all of the metabolites are detected at femtomolar concentrations, and therefore the sample volumes required for this analysis are small. Our method has a potential to be adopted for measuring concentrations of progesterone metabolites as novel biomarkers in tissue samples and physiological fluids.

## Supporting Information

S1 Fig ^1^H NMR characterization of (20S)-pregn-4-ene-3β,20-diol. (PDF)

S2 Fig MS characterization of (20S)-pregn-4-ene-3β,20-diol. (PDF)

S3 Fig HPLC characterization of (20S)-pregn-4-ene-3β,20-diol. (PDF)

S4 Fig ^1^H NMR characterization of (20S)-pregn-4-ene-3α,20-diol. (PDF)

S5 Fig MS characterization of (20S)-pregn-4-ene-3α,20-diol. (PDF)

S6 Fig HPLC characterization of (20S)-pregn-4-ene-3α,20-diol. (PDF)

S7 Fig ^1^H NMR characterization of (20R)-pregn-4-ene-3β,20-diol. (PDF)

S8 Fig MS characterization of (20R)-pregn-4-ene-3β,20-diol. (PDF)

S9 Fig HPLC characterization of (20R)-pregn-4-ene-3β,20-diol. (PDF)

S10 Fig ^1^H NMR characterization of (20R)-pregn-4-ene-3α,20-diol. (PDF)

S11 Fig MS characterization of (20R)-pregn-4-ene-3α,20-diol. (PDF)

S12 Fig HPLC characterization of (20R)-pregn-4-ene-3α,20-diol. (PDF)

S13 Fig Blank sample: solvent with 100 ng/mL of testosterone. (TIF)
