# Supplementary figures and images for: Combined Liquid Chromatography–Tandem Mass Spectrometry Analysis of Progesterone Metabolites
Source: PLoS One. 2015 Feb 13;10(2):e0117984. doi: 10.1371/journal.pone.0117984 (PMC4332660; doi:10.1371/journal.pone.0117984)

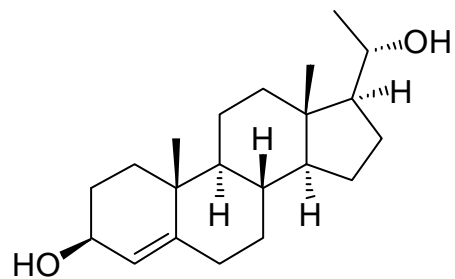

3β,20α-P4

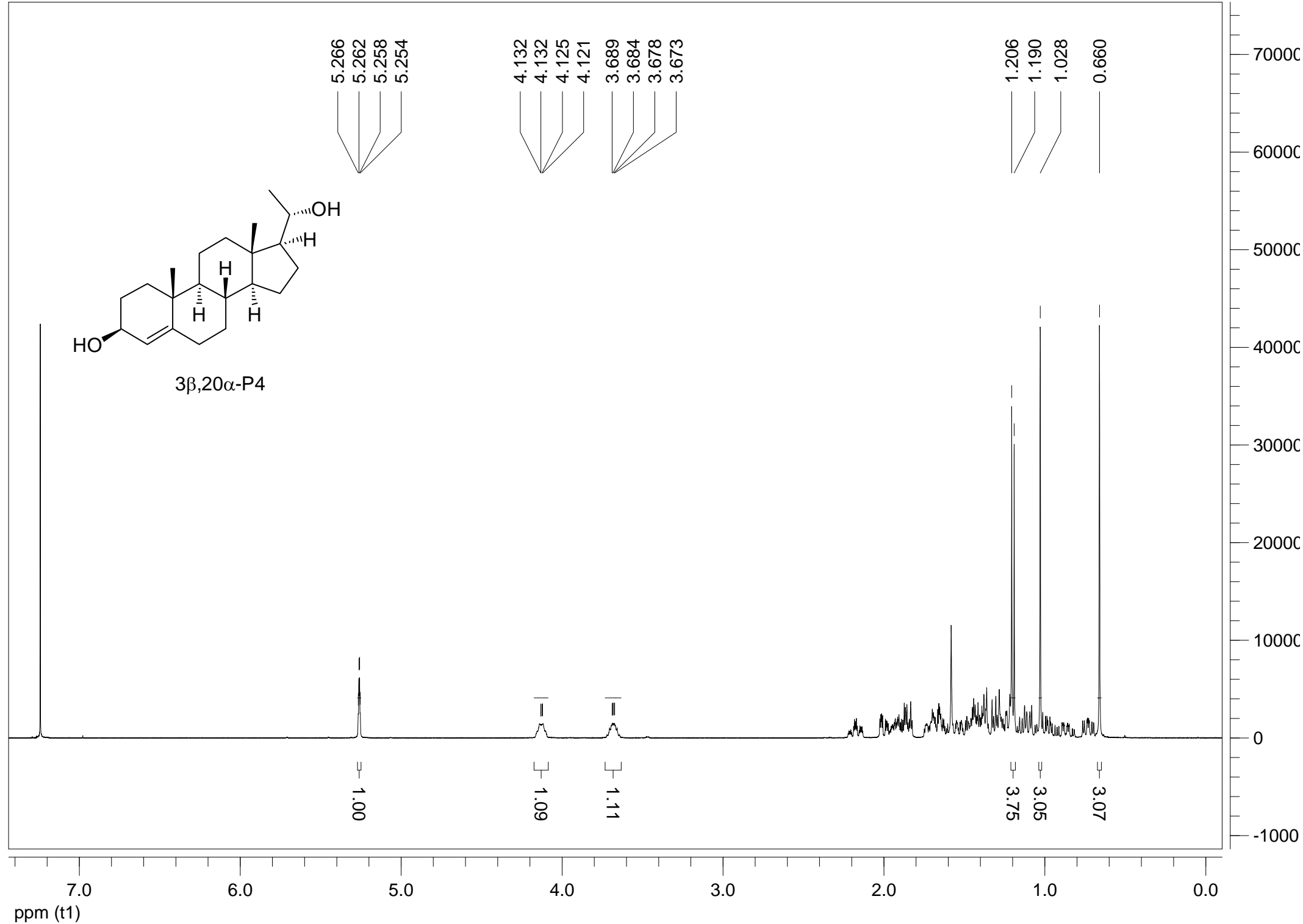

Supplement: S1 Fig — (PDF) [file pone.0117984.s001.pdf]

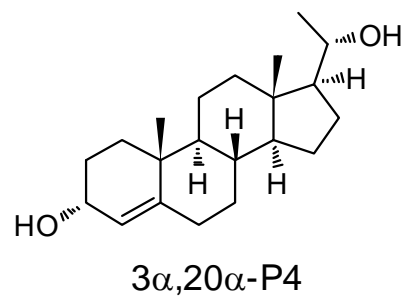

5.474  
5.470  
5.462  
5.457

4.079  
4.073  
4.068  
3.722  
3.707  
3.688

1.232  
1.217  
0.984  
0.690

10000

50000

0

1.00

1.04

1.11

3.49

3.00

3.00

ppm (t1)

Supplement: S4 Fig — (PDF) [file pone.0117984.s004.pdf]

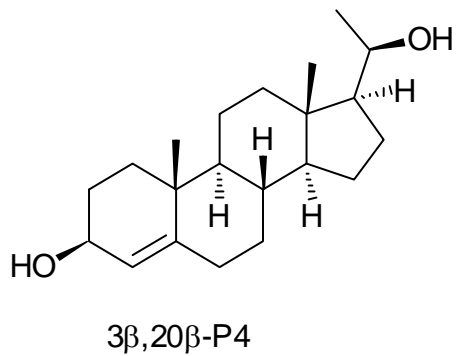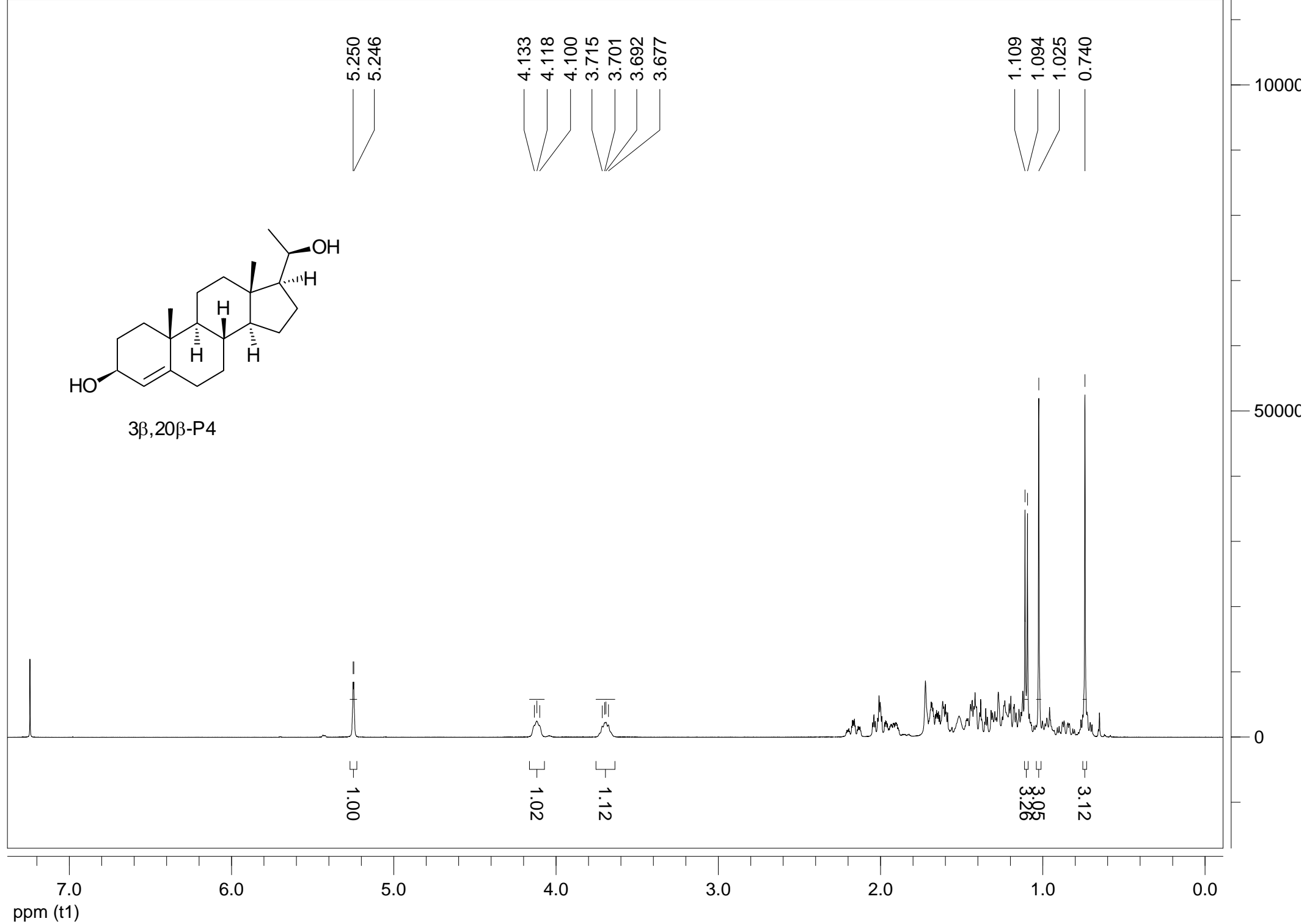

Supplement: S7 Fig — (PDF) [file pone.0117984.s007.pdf]

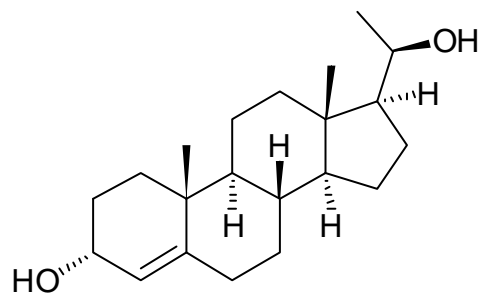

3 $\alpha$ ,20 $\beta$ -P4

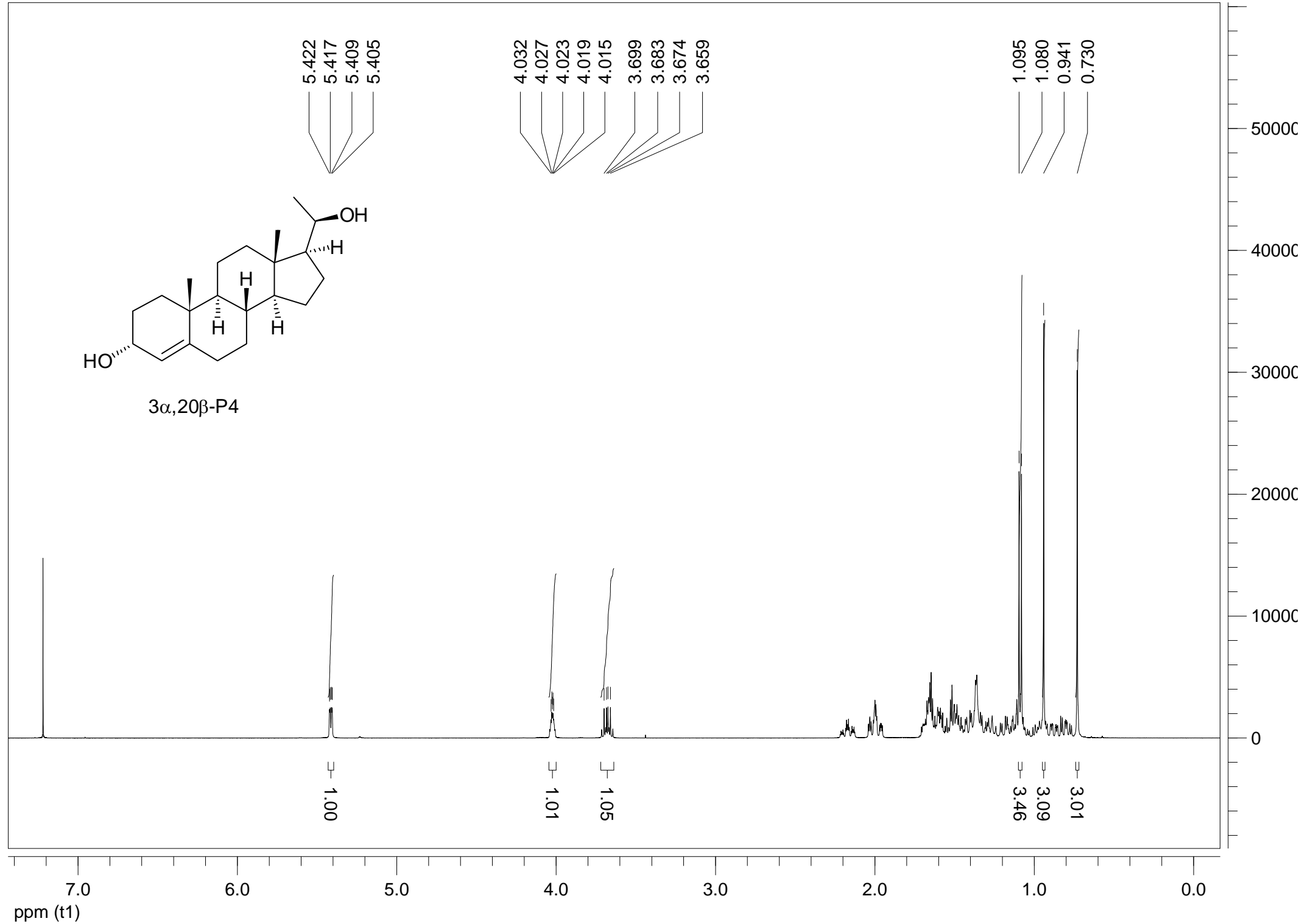

Supplement: S10 Fig — (PDF) [file pone.0117984.s010.pdf]

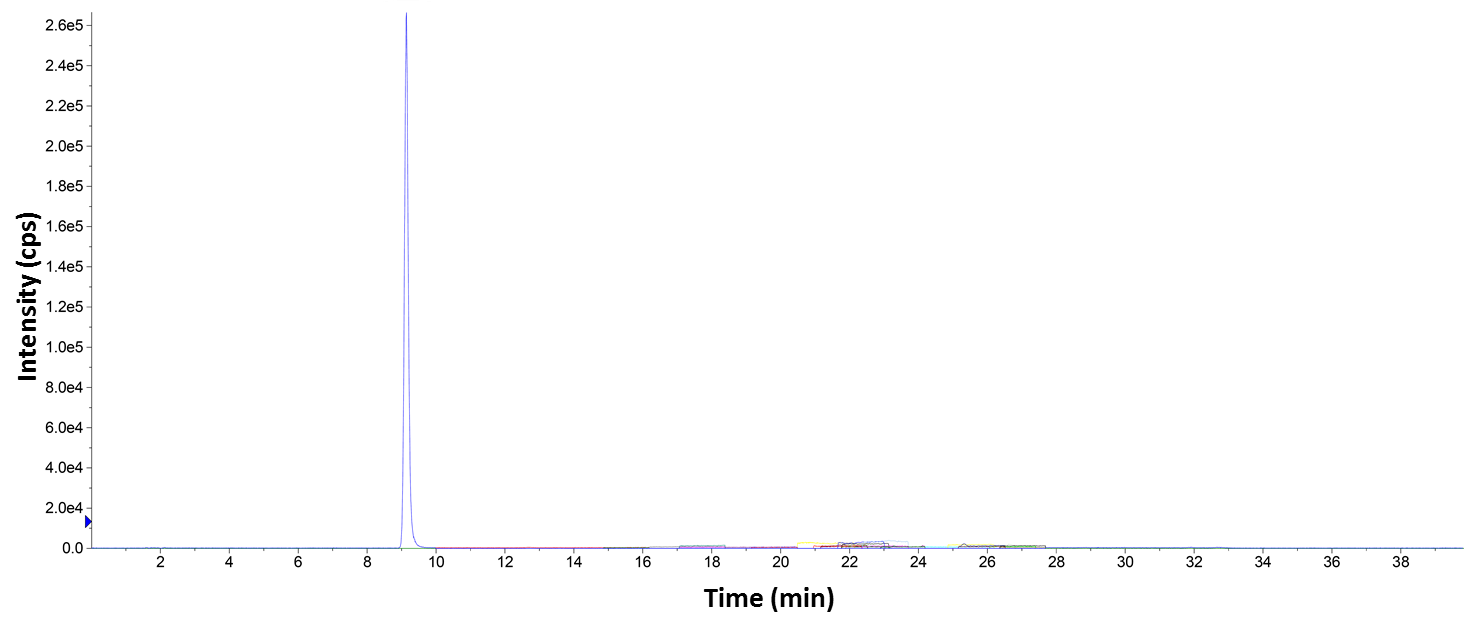

Supplement: S13 Fig — (TIF) [file pone.0117984.s013.tif]
