# Supplementary material for: Combined Liquid Chromatography–Tandem Mass Spectrometry Analysis of Progesterone Metabolites
Source: PLoS One. 2015 Feb 13;10(2):e0117984. doi: 10.1371/journal.pone.0117984 (PMC4332660; doi:10.1371/journal.pone.0117984)
Supplement: S2 Fig — (PDF) [file pone.0117984.s002.pdf]

Single Mass Analysis
Tolerance = 10.0 PPM / DBE: min = -1.5, max = 50.0
Element prediction: Off
Number of isotope peaks used for i-FIT = 3

Monoisotopic Mass, Even Electron Ions
682 formula(e) evaluated with 4 results within limits (all results (up to 1000) for each mass)
Elements Used:
C: 0-100 H: 0-100 N: 0-20 O: 0-20
JC_SI-P4 3beta 20alfa 10 (0.461) Cm (7:11-27:31)
1: TOF MS ES+

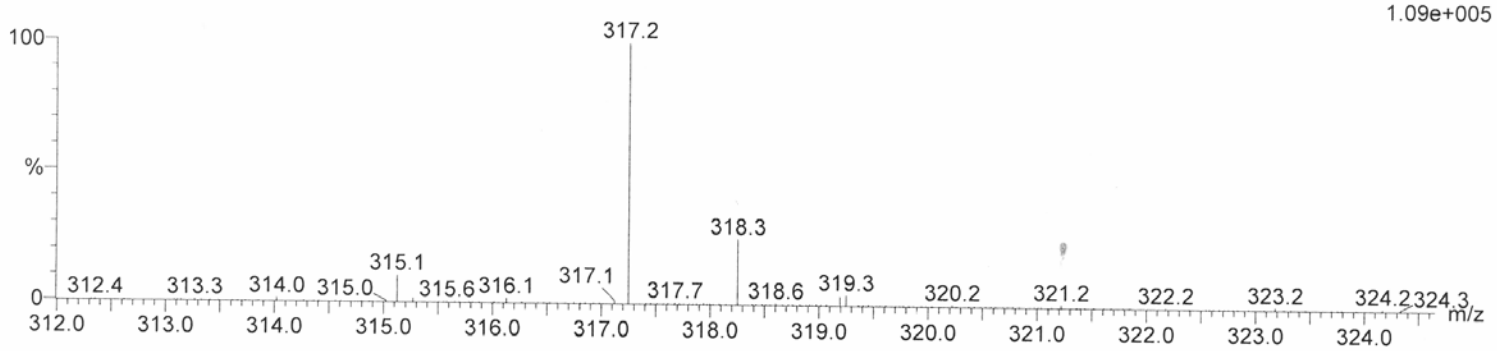

Minimum:				-1.5			
Maximum:		5.0	10.0	50.0			
Mass	Calc. Mass	mDa	PPM	DBE	i-FIT	i-FIT (Norm)	Formula
317.2484	317.2481	0.3	0.9	5.5	436.8	0.0	C21 H33 O2
	317.2454	3.0	9.5	6.5	440.4	3.6	C17 H29 N6
	317.2486	-0.2	-0.6	-1.5	444.8	8.0	C6 H29 N12 O3
	317.2459	2.5	7.9	-0.5	446.9	10.1	C2 H25 N18 O

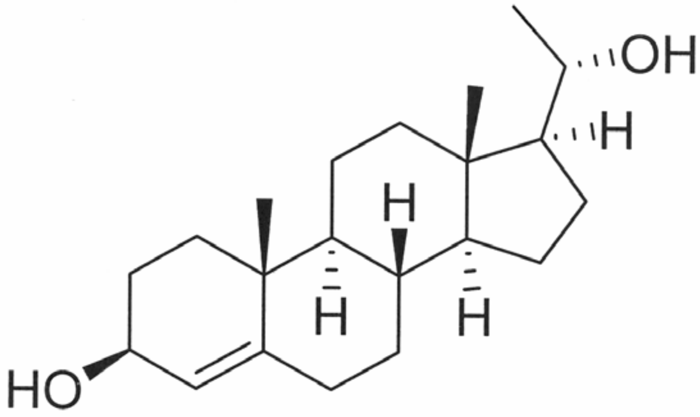

3β,20α-P4
