# Supplementary material for: Combined Liquid Chromatography–Tandem Mass Spectrometry Analysis of Progesterone Metabolites
Source: PLoS One. 2015 Feb 13;10(2):e0117984. doi: 10.1371/journal.pone.0117984 (PMC4332660; doi:10.1371/journal.pone.0117984)
Supplement: S3 Fig — (PDF) [file pone.0117984.s003.pdf]

Data File C:\CHEM32\1\DATA\JC_SI_P4\JC_SI_P4 2013-01-18 14-53-00\002-0201.D
Sample Name: peak 1

===

Acq. Operator : Jozko
Seq. Line : 2
Acq. Instrument : HPLC
Location : Vial 2
Injection Date : 1/18/2013 3:08:56 PM
Inj : 1
Inj Volume : 10 µl
Acq. Method : C:\Chem32\1\DATA\JC_SI_P4\JC_SI_P4 2013-01-18 14-53-00\JC_SI_P4_MECN.M
Last changed : 1/17/2013 6:29:42 PM by Jozko
Analysis Method : C:\CHEM32\1\DATA\JC_SI_P4\JC_SI_P4 2013-01-18 14-53-00\002-0201.D\DA.M (JC_SI_P4_MECN.M)
Last changed : 1/17/2013 6:29:42 PM by Jozko
Method Info : Analiza derivatov po redukciji progesterona
Voda / MeCN = 45 /55
Kolona: Agilent Eclipse Plus C16 150 x 4.6 mm, 5 um
pretok 1.2 ml/min, 25 °C, V = 10 ul, 206 nm + 240 nm, 12 min

Sample Info : P4-3beta, 20alpha

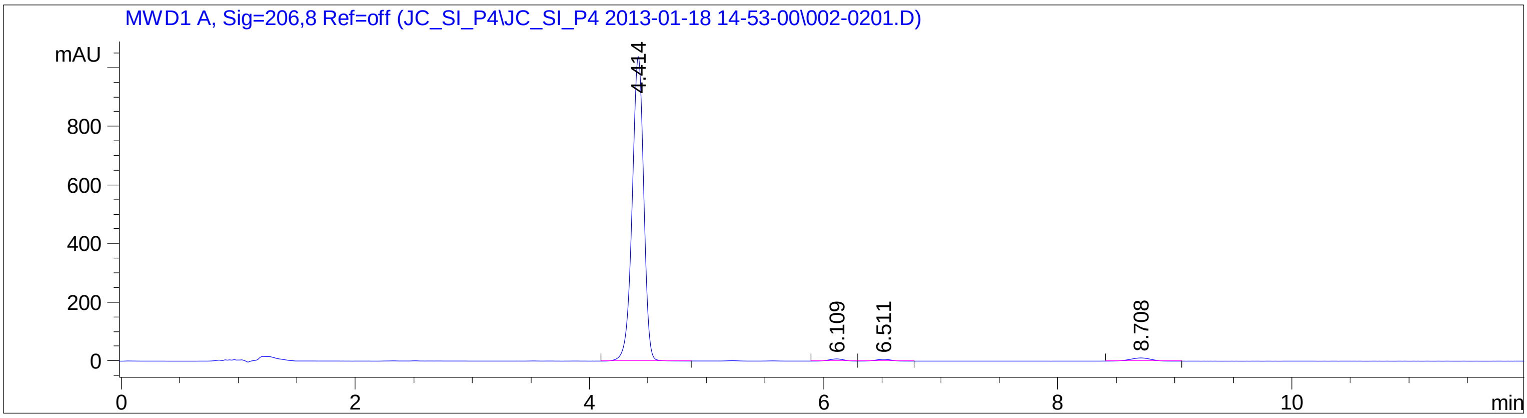

===

Area Percent Report

===

Sorted By : Signal
Multiplier : 1.0000
Dilution : 1.0000
Do not use Multiplier & Dilution Factor with ISTDs

Signal 1: MWD1 A, Sig=206,8 Ref=off

Peak #	RetTime [min]	Type	Width [min]	Area [mAU*s]	Height [mAU]	Area %
1	4.414	BB	0.1060	7115.73145	1038.00623	96.4377
2	6.109	BV	0.1421	66.88280	7.35368	0.9064
3	6.511	VB	0.1484	58.62856	6.08552	0.7946
4	8.708	BB	0.1980	137.33667	10.82822	1.8613

Totals : 7378.57948 1062.27365

===

*** End of Report ***
