# Supplementary material for: Combined Liquid Chromatography–Tandem Mass Spectrometry Analysis of Progesterone Metabolites
Source: PLoS One. 2015 Feb 13;10(2):e0117984. doi: 10.1371/journal.pone.0117984 (PMC4332660; doi:10.1371/journal.pone.0117984)
Supplement: S6 Fig — (PDF) [file pone.0117984.s006.pdf]

Data File C:\CHEM32\1\DATA\JC_SI_P4\JC_SI_P4 2013-01-18 14-53-00\003-0301.D
Sample Name: peak 2

===

Acq. Operator : Jozko   Seq. Line : 3
Acq. Instrument : HPLC   Location : Vial 3
Injection Date : 1/18/2013 3:23:01 PM   Inj : 1
Inj Volume : 10 µl
Acq. Method : C:\Chem32\1\DATA\JC_SI_P4\JC_SI_P4 2013-01-18 14-53-00\JC_SI_P4_MECN.M
Last changed : 1/17/2013 6:29:42 PM by Jozko
Analysis Method : C:\CHEM32\1\DATA\JC_SI_P4\JC_SI_P4 2013-01-18 14-53-00\003-0301.D\DA.M (JC_SI_P4_MECN.M)
Last changed : 1/17/2013 6:29:42 PM by Jozko
Method Info : Analiza derivatov po redukciji progesterona
Voda / MeCN = 45 /55
Kolona: Agilent Eclipse Plus C16 150 x 4.6 mm, 5 um
pretok 1.2 ml/min, 25 °C, V = 10 ul, 206 nm + 240 nm, 12 min

Sample Info : P4-3alpha, 20alpha

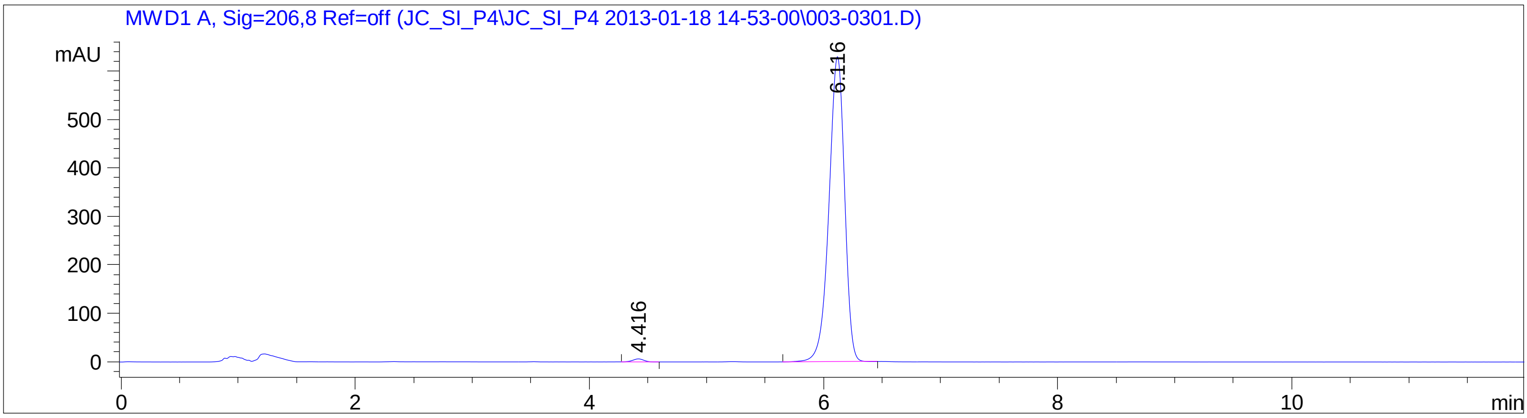

===

Area Percent Report

===

Sorted By : Signal
Multiplier : 1.0000
Dilution : 1.0000
Do not use Multiplier & Dilution Factor with ISTDs

Signal 1: MWD1 A, Sig=206,8 Ref=off

Peak #	RetTime [min]	Type	Width [min]	Area [mAU*s]	Height [mAU]	Area %
1	4.416	BB	0.1013	41.42162	6.24817	0.7030
2	6.116	BB	0.1444	5850.99707	629.91925	99.2970

Totals : 5892.41869 636.16742

===

*** End of Report ***
