# Supplementary material for: Combined Liquid Chromatography–Tandem Mass Spectrometry Analysis of Progesterone Metabolites
Source: PLoS One. 2015 Feb 13;10(2):e0117984. doi: 10.1371/journal.pone.0117984 (PMC4332660; doi:10.1371/journal.pone.0117984)
Supplement: S8 Fig — (PDF) [file pone.0117984.s008.pdf]

Single Mass Analysis

Tolerance = 10.0 PPM / DBE: min = -1.5, max = 50.0
Element prediction: Off
Number of isotope peaks used for i-FIT = 3

Monoisotopic Mass, Even Electron Ions
682 formula(e) evaluated with 4 results within limits (all results (up to 1000) for each mass)
Elements Used:
C: 0-100 H: 0-100 N: 0-20 O: 0-20
JC_SI-P4 3beta 20beta 25 (1.161) Cm (22:27)
1: TOF MS ES+

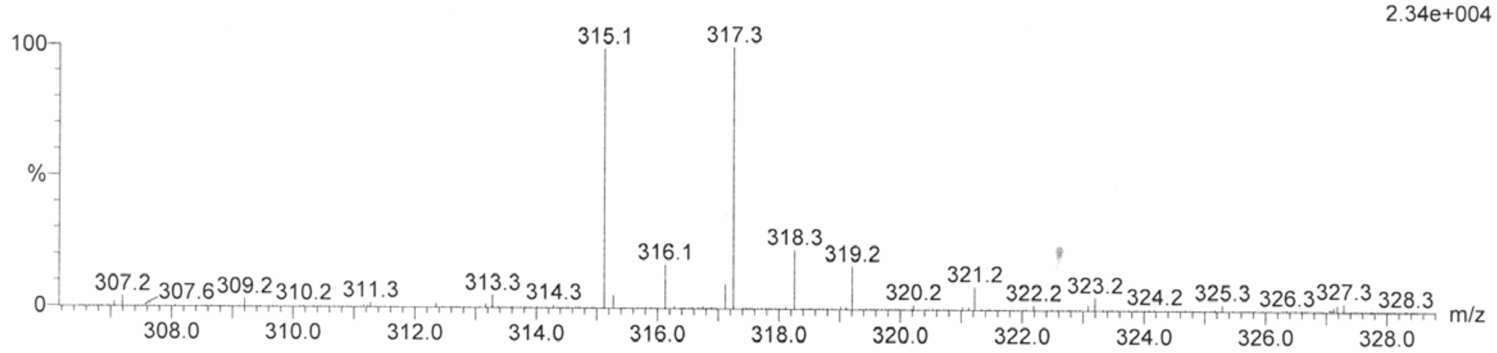

Mass	Calc. Mass	mDa	PPM	DBE	i-FIT	i-FIT (Norm)	Formula
Minimum:				-1.5			
Maximum:		5.0	10.0	50.0			
317.2485	317.2481	0.4	1.3	5.5	302.7	0.9	C21 H33 O2
	317.2454	3.1	9.8	6.5	302.9	1.1	C17 H29 N6
	317.2486	-0.1	-0.3	-1.5	303.6	1.9	C6 H29 N12 O3
	317.2459	2.6	8.2	-0.5	303.8	2.0	C2 H25 N18 O

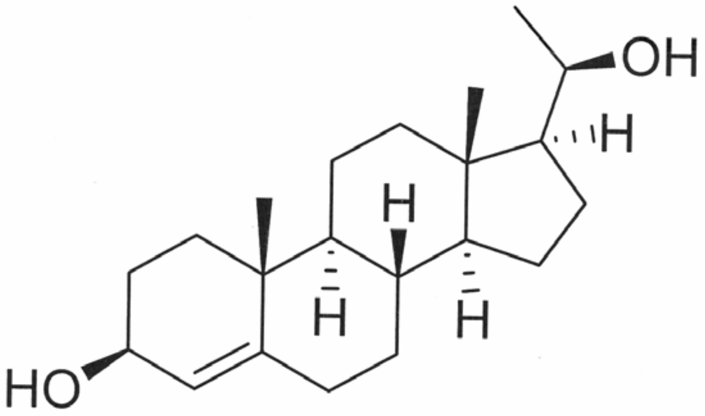

3β,20β-P4
