# Supplementary material for: Combined Liquid Chromatography–Tandem Mass Spectrometry Analysis of Progesterone Metabolites
Source: PLoS One. 2015 Feb 13;10(2):e0117984. doi: 10.1371/journal.pone.0117984 (PMC4332660; doi:10.1371/journal.pone.0117984)
Supplement: S9 Fig — (PDF) [file pone.0117984.s009.pdf]

Data File C:\CHEM32\1\DATA\JC_SI_P4\JC_SI_P4 2013-01-18 14-53-00\004-0401.D
Sample Name: peak 3

===

Acq. Operator : Jozko　　Seq. Line : 4
Acq. Instrument : HPLC　　Location : Vial 4
Injection Date : 1/18/2013 3:37:08 PM　　Inj : 1
Inj Volume : 10 µl
Acq. Method : C:\Chem32\1\DATA\JC_SI_P4\JC_SI_P4 2013-01-18 14-53-00\JC_SI_P4_MECN.M
Last changed : 1/17/2013 6:29:42 PM by Jozko
Analysis Method : C:\CHEM32\1\DATA\JC_SI_P4\JC_SI_P4 2013-01-18 14-53-00\004-0401.D\DA.M (JC_SI_P4_MECN.M)
Last changed : 1/17/2013 6:29:42 PM by Jozko
Method Info : Analiza derivatov po redukciji progesterona
Voda / MeCN = 45 /55
Kolona: Agilent Eclipse Plus C16 150 x 4.6 mm, 5 um
pretok 1.2 ml/min, 25 °C, V = 10 ul, 206 nm + 240 nm, 12 min

Sample Info : P4-3beta, 20beta

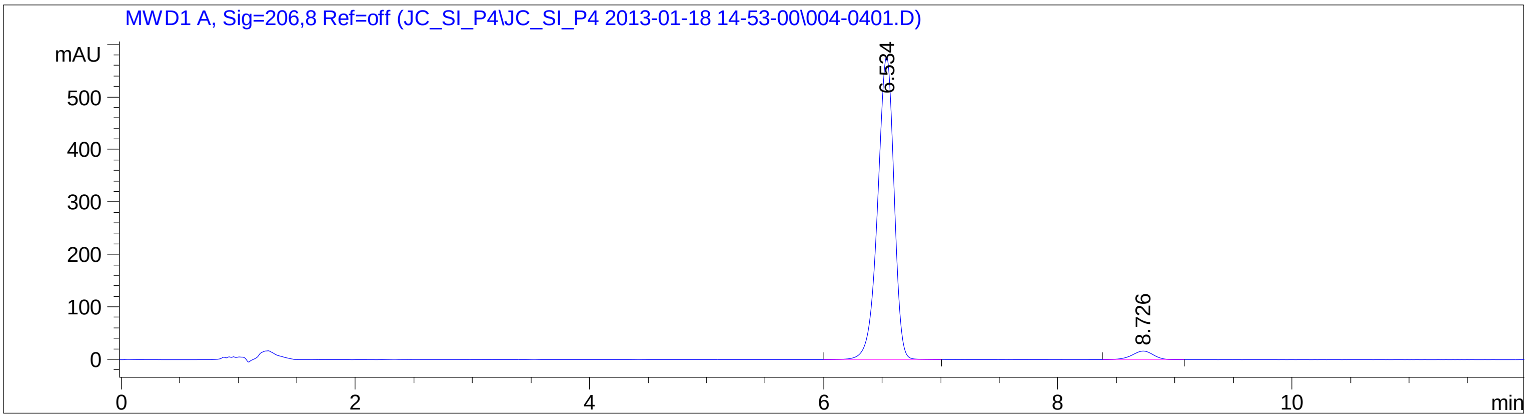

===

Area Percent Report

===

Sorted By : Signal
Multiplier : 1.0000
Dilution : 1.0000
Do not use Multiplier & Dilution Factor with ISTDs

Signal 1: MWD1 A, Sig=206,8 Ref=off

Peak #	RetTime [min]	Type	Width [min]	Area [mAU*s]	Height [mAU]	Area %
1	6.534	BB	0.1517	5622.65771	576.86383	96.4201
2	8.726	BB	0.2014	208.75832	16.30374	3.5799

Totals : 5831.41603 593.16757

===

*** End of Report ***
