# Supplementary material for: Combined Liquid Chromatography–Tandem Mass Spectrometry Analysis of Progesterone Metabolites
Source: PLoS One. 2015 Feb 13;10(2):e0117984. doi: 10.1371/journal.pone.0117984 (PMC4332660; doi:10.1371/journal.pone.0117984)
Supplement: S11 Fig — (PDF) [file pone.0117984.s011.pdf]

Single Mass Analysis

Tolerance = 10.0 PPM / DBE: min = -1.5, max = 50.0
Element prediction: Off
Number of isotope peaks used for i-FIT = 3

Monoisotopic Mass, Even Electron Ions
682 formula(e) evaluated with 3 results within limits (all results (up to 1000) for each mass)
Elements Used:
C: 0-100 H: 0-100 N: 0-20 O: 0-20
JC_SI-P4 3alfa 20beta 5 (0.240) Cm (2:12)
1: TOF MS ES+

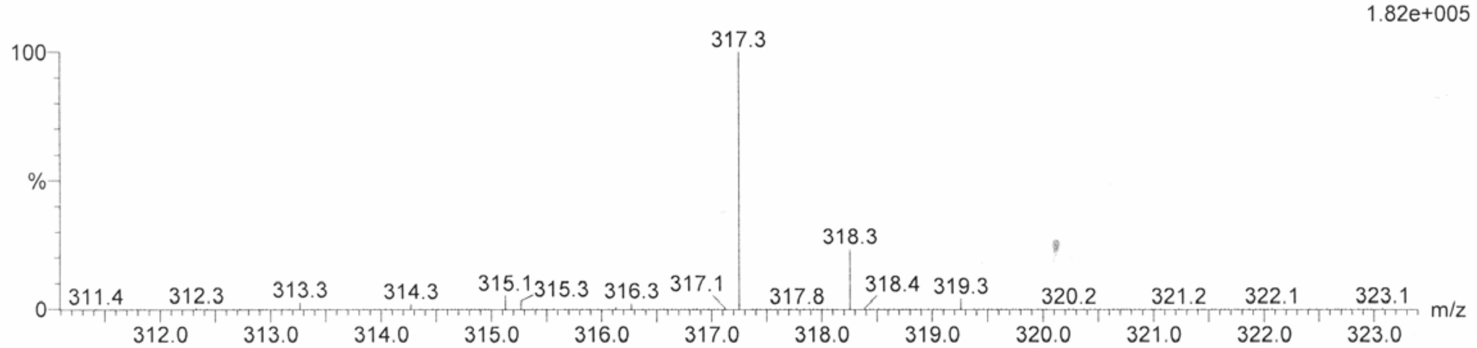

Minimum:				-1.5			
Maximum:		5.0	10.0	50.0			

Mass	Calc. Mass	mDa	PPM	DBE	i-FIT	i-FIT (Norm)	Formula
317.2489	317.2481	0.9	2.8	5.5	390.4	0.0	C21 H33 O2
	317.2486	0.4	1.3	-1.5	400.4	10.1	C6 H29 N12 O3
	317.2459	3.1	9.8	-0.5	402.9	12.6	C2 H25 N18 O

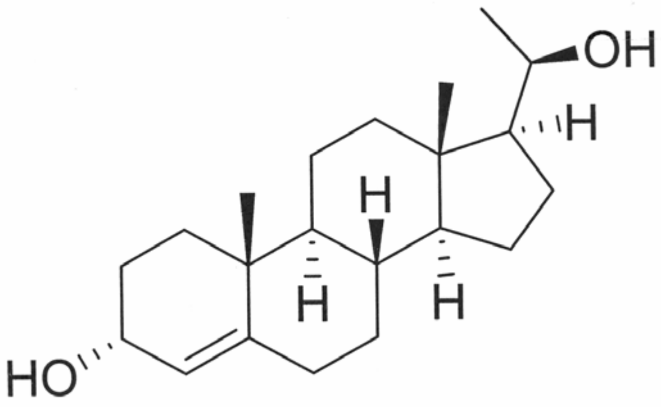

3α,20β-P4
