# Supplementary material for: Combined Liquid Chromatography–Tandem Mass Spectrometry Analysis of Progesterone Metabolites
Source: PLoS One. 2015 Feb 13;10(2):e0117984. doi: 10.1371/journal.pone.0117984 (PMC4332660; doi:10.1371/journal.pone.0117984)
Supplement: S12 Fig — (PDF) [file pone.0117984.s012.pdf]

===

Acq. Operator : Jozko Seq. Line : 5
Acq. Instrument : HPLC Location : Vial 5
Injection Date : 1/18/2013 3:51:13 PM Inj : 1
Inj Volume : 10 µl
Acq. Method : C:\Chem32\1\DATA\JC_SI_P4\JC_SI_P4 2013-01-18 14-53-00\JC_SI_P4_MECN.M
Last changed : 1/17/2013 6:29:42 PM by Jozko
Analysis Method : C:\CHEM32\1\DATA\JC_SI_P4\JC_SI_P4 2013-01-18 14-53-00\005-0501.D\DA.M (JC_SI_P4_MECN.M)
Last changed : 1/17/2013 6:29:42 PM by Jozko
Method Info : Analiza derivatov po redukciji progesterona
Voda / MeCN = 45 /55
Kolona: Agilent Eclipse Plus C16 150 x 4.6 mm, 5 um
pretok 1.2 ml/min, 25 °C, V = 10 ul, 206 nm + 240 nm, 12 min

Sample Info : P4-3alpha, 20abeta

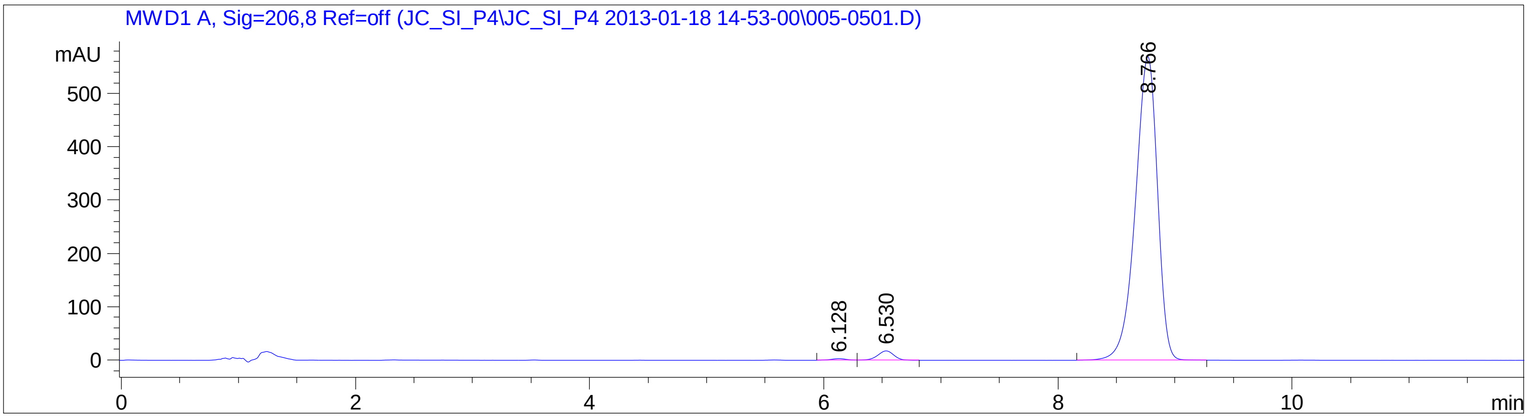

===

Area Percent Report

===

Sorted By : Signal
Multiplier : 1.0000
Dilution : 1.0000
Do not use Multiplier & Dilution Factor with ISTDs

Signal 1: MWD1 A, Sig=206,8 Ref=off

Peak #	RetTime [min]	Type	Width [min]	Area [mAU*s]	Height [mAU]	Area %
1	6.128	BV	0.1452	28.11866	3.05937	0.3667
2	6.530	VB	0.1486	169.58022	17.57164	2.2118
3	8.766	BB	0.2030	7469.30615	569.59607	97.4214

Totals : 7667.00503 590.22708

===

*** End of Report ***
